# Potassium Ferrite for Biomedical Applications

**DOI:** 10.3390/ma16103880

**Published:** 2023-05-22

**Authors:** João P. F. Carvalho, Tânia Vieira, Jorge Carvalho Silva, Paula I. P. Soares, Nuno M. Ferreira, Carlos O. Amorim, Sílvia Soreto Teixeira, Manuel P. F. Graça

**Affiliations:** 1 i3N and Department of Physics, University of Aveiro, 3810-193 Aveiro, Portugal; jpfc@ua.pt (J.P.F.C.); nmferreira@ua.pt (N.M.F.); silvia.soreto@ua.pt (S.S.T.); 2 i3N/CENIMAT, Physics Department, NOVA School of Science and Technology, Campus de Caparica, NOVA University Lisbon, 2829-516 Caparica, Portugal; ts.vieira@fct.unl.pt (T.V.); jcs@fct.unl.pt (J.C.S.); 3 i3N/CENIMAT, Materials Science Department, NOVA School of Science and Technology, Campus de Caparica, NOVA University Lisbon, 2829-516 Caparica, Portugal; pi.soares@fct.unl.pt; 4 CICECO-Aveiro Institute of Materials and Department of Physics, University of Aveiro, 3810-193 Aveiro, Portugal; amorim5@ua.pt

**Keywords:** ferrites, sol-gel processes, magnetic measurements (VSM and SQUID), specific absorption rate (SAR), X-ray diffraction, cellular viability

## Abstract

Ferrites have been widely studied for their use in the biomedical area, mostly due to their magnetic properties, which gives them the potential to be used in diagnostics, drug delivery, and in treatment with magnetic hyperthermia, for example. In this work, KFeO_2_ particles were synthesized with a proteic sol-gel method using powdered coconut water as a precursor; this method is based on the principles of green chemistry. To improve its properties, the base powder obtained was subjected to multiple heat treatments at temperatures between 350 and 1300 °C. The samples obtained underwent structural, morphological, biocompatibility, and magnetic characterization. The results show that upon raising the heat treatment temperature, not only is the wanted phase detected, but also the secondary phases. To overcome these secondary phases, several different heat treatments were carried out. Using scanning electron microscopy, grains in the micrometric range were observed. Saturation magnetizations between 15.5 and 24.1 emu/g were observed for the samples containing KFeO_2_ with an applied field of 50 kOe at 300 K. From cellular compatibility (cytotoxicity) assays, for concentrations up to 5 mg/mL, only the samples treated at 350 °C were cytotoxic. However, the samples containing KFeO_2_, while being biocompatible, had low specific absorption rates (1.55–5.76 W/g).

## 1. Introduction

More than half a century ago, magnetic nanoparticles were used for the first time in magnetic hyperthermia. The used technique is the same one used today: the particles are exposed to an alternate magnetic field to transfer heat [1]. This transfer of heat is defined by the specific absorption rate (SAR). Nanoparticles, particles defined as having a size between 1 and 100 nm [2], particularly magnetic nanoparticles, have been one of the objects of study in the biomedical area. These kinds of particles can be used for diagnostics, such as in imaging, to the treatment of patients, varying from drug delivery to magnetic hyperthermia [3,4]. When it comes to eliminating cancer cells, iron oxide-based nanoparticles have been widely studied [4]. Removing cancer cells through magnetic hyperthermia demands that the particles have a certain size to prevent them from blocking small capillaries or being mechanically filtered. Taking this account, they should have an ideal size, which should be between 5 and 100 nm [5]. Although presenting magnetic properties with potential application in the biomedical field, ferrites of nickel (Ni), manganese (Mn), cobalt (Co), or zinc (Zn), have an inherent toxicity that makes them more difficult to apply in this area [6], which means they need a coating. According to the literature [6], due to their magnetic properties, ferrite nanoparticles have the potential to be used in magnetic hyperthermia. When applied to medicine, this therapy consists of exposing part of the body to higher temperatures than the normal body temperature, which has a therapeutic effect.

Potassium ferrite is a monocrystalline solid with an orthorhombic crystalline structure; it is a hygroscopic compound that tends to absorb humidity, making it unstable. For this reason, it is not extensively reported in the literature [7]. Potassium ferrite is a compound that is sensitive to ambient conditions. The existence of moisture and carbon dioxide in ambient air originates from K^+^ extraction in KFeO_2_, which will react with the H_2_O and CO_2_ in the air, which is where K_2_CO_3_·1.5 H_2_O originates, and in addition, a phase evolution in KFeO_2_ originates. This reaction can be reverted by high-temperature calcination, which gives this material potential to be applied in high-temperature rechargeable batteries [8].

Its magnetic characteristic is a point of conflict in the literature, having been reported as having an antiferromagnetic [7,9] and a ferromagnetic behaviour [6,10]. This last behaviour, in a superparamagnetic state, could provide the potential for the application of these nanoparticles in the biomedical area [6,10]. For this kind of application, the particles must be subjected to a biocompatibility assay.

The main purposes of this work were the synthesis, physical characterization, and evaluation of potential in the magnetic hyperthermia of potassium ferrite particles. Since this ferrite has in its composition a non-toxic metal, we would expect that it would present bigger biocompatibility than the previously described ferrites, as it is one of the major reasons for the study of this material. Besides that, potassium ferrite is not vastly studied in the literature, and it is a study of major interest, as there could be significant applications that have not been explored yet. Overall, it was the aim of this work to provide insights into potassium ferrite produced by an eco-friendly method in biomedicine, specifically for magnetic hyperthermia treatments.

These particles were synthesized with a novel sol-gel method through a proteic route using metal nitrates as raw materials. Powdered coconut water (PCW) was used as a precursor, respecting green chemistry principles, such as the prevention of waste, as very little was created in the synthesis; the use of less hazardous chemicals; and the use of a renewable precursor (PCW), turning this method into an eco-friendly route [11], as opposed to other routes that use ethylene glycol and citric acid [12,13,14]. This method has already been used in the synthesis of nanoparticles and ceramic materials, such as BaFe_12_O_19_ [15] and SrFe_12_O_19_ [16].

With this work, we intended to evaluate the influence of the heat treatment’s parameters (time and temperature) on the formation of the potassium ferrite phase, the grain size, the magnetic behaviour, their biocompatibility, and the specific absorption rate of the particles (SAR).

## 2. Materials and Methods

### 2.1. Potassium Ferrite Powder Preparation

The potassium ferrite powder was prepared using a proteic sol-gel route. To obtain the ferrite powder, iron (III) nitrate (Fe(NO_3_)_3_·9H_2_O; Fluka, Buchs, Switzerland; ≥99%) and potassium nitrate (KNO_3_; Aldrich, Fallavier, France; ≥95%) were used as raw materials with a molar ratio of 1 mol K^+^: 1 mol Fe^+2^. The precursor of the reaction, PCW, was used to prepare a solution with a concentration of 0.59 mol/dm^3^, which is the critical micellar concentration. The raw materials were mixed with a magnetic stirrer using the following conditions: (i) T = 80 °C, ∆t= 2 h, and (ii) T = 100 °C, ∆t= 2.5 h, to obtain a viscous gel. With the intent of removing all the solvent and to obtain a dry powder, a heat treatment at 350 °C was made for an hour. This dried powder shaped into a disk form (13 mm diameter) was heat treated with temperatures between 350 and 1300 °C using a 5 °C/min heating rate. A uniaxial hydraulic press with a stainless steel mould was employed, and a 15 $tnf/{cm}^{2}$ was used for 3 min.

### 2.2. Structural and Morphological Characterization

The thermal differential and thermogravimetric analyses (DTA/TGA) were made with Hitachi STA7300 equipment in an oxidative atmosphere (140 mL N_2_ and 60 mL O_2_) from room temperature up to 1300 °C using a heating rate of 5 °C/min, allowing it to determine the heat treatment temperatures to apply.

Moving on to the structural characterization, the powder X-ray diffraction (XRD) pattern data were obtained using an Empyrean diffractometer (CuK α radiation, λ ≈ 1.54060 Å) operating at 45 kV and 40 mA. Intensity data were collected with the step counting method (step 0.02°s^−1^) in the 2θ angle range of 10–60°. The identification of the crystalline phases was done using the X’Pert HighScore PANalytical software equipped with the database of the Joint Committee for Powder Diffraction Standards–International Center for Diffraction Data (JCPDS).

Micro-Raman spectroscopy was another technique that was performed utilizing a Jobin–Yvon spectrometer. The measurements were performed at room temperature using a setup with backscattering geometry. A microscope objective (50×) was used to focus the laser exciting light (λ = 532 nm) onto the sample (spot diameter < 0.8 µm). A filter was employed to remove the plasma lines.

To complement the previous analysis, the Fourier Transform Infrared Spectroscopy (FTIR) technique was performed using a Perkin Elmer FTIR System Spectrum BX Spectrometer equipped with a single horizontal Golden Gate ATR cell. All data were recorded at room temperature in the range of 4000 to 600 cm^−1^ by accumulating 32 scans with a resolution of 4 cm^−1^.

The samples’ morphology was studied with scanning electron microscopy (SEM) using a Vega 3 TESCAN microscope. Conductivity was assured by carbon deposition on the samples. Average grain size was obtained using the ImageJ software.

### 2.3. Cytotoxicity Analysis

The cytotoxicity assays were made according to standard ISO-10993 (Biological evaluation of medical devices–Part 5: Tests for in vitro cytotoxicity). The tests were made using the extract method, and Saos-2 cells line were provided by the American Type Culture Collection (ATCC HTB-85). The protocols for expansion and maintenance were followed according to the provider recommendations. This lineage of cells was obtained from the bone of an 11-year-old white female osteosarcoma patient (ATCC HTB-85). The extract was produced using, initially, a concentration of 20 mg/mL of each sample with different heat treatments. Each sample was placed in 1 mL of McCoy-5A medium (Sigma Aldrich, Fallavier, France) at 37 °C for 48 h. The Saos-2 cells were seeded in 96-well plates at a density of 30,000 cells/${cm}^{2}$, having been grown in McCoy-5A supplemented with sodium bicarbonate (2.2 g/L), 10% fetal bovine serum (Biowest, Nuaillé, France), and 1% Penicillin-Streptomycin (Life Technologies, Zuid, Holland) at 37 °C in a humidified environment of 5% $CO_{2}$ for 24 h. The extract was diluted in four concentrations: 10, 5, 2.5, and 1.125 mg/mL. Each of the concentrations was tested 4 times. A negative control with cells in a normal environment and a positive control with cells treated with 10% DMSO (a cytotoxic compound) were set up. After 48 h of incubation, the medium was removed from each well and replaced with a resazurin solution containing 50% of the complete medium and 50% of a 0.04 mg/mL resazurin solution in phosphate-buffered saline (PBS). After 3 h of incubation at 37 °C and 5% $CO_{2}$, the absorbance was measured at 570 and 600 nm. Cell viability is given as a percentage of viable cells in the samples to test relative to the negative control:

$$Celular\;Viability\left(\%\right)=\frac{Sample’s\;Absorbance}{Control\;Cells\;Absorbance}\times 100\%$$

### 2.4. Magnetic Characterization

The magnetic measurements were made using a vibrating sample magnetometer (VSM) model Cryofree from Cryogenic. The magnetic curves were obtained for the temperatures of 5 and 300 K using a magnetic field (H) of up to 50 kOe [17].

To further analyse the magnetic properties of the sample with the highest percentage of potassium ferrite (1000 °C 2HT), a Quantum Design MPMS3 SQUID-VSM was used to perform magnetization measurements as a function of the applied magnetic field up to 70 kOe, at 5, 300, and 380 K. Sample geometry effects were corrected using the methodology reported in [18]. To have more accurate coercive fields, the remanent fields from the superconducting coil with calibration measurements were also corrected using a reference Pd sample from NIST between −2 kOe and +2 kOe.

Magnetic hyperthermia (MHT) measurements were performed to determine the specific absorption rate (SAR) by using DM100 Series equipment from nB nanoscale Biomagnetics. The SAR was measured for a concentration of 10 mg/mL of the sample using an alternating external magnetic field of 24 kA/m with a frequency of 418.5 kHz for 10 min. Before each measurement, samples were immersed in 1 mL of ultrapure water and ultra-sonicated.

## 3. Results and Discussion

### 3.1. Thermal and Structural Analysis

**Figure 1:**
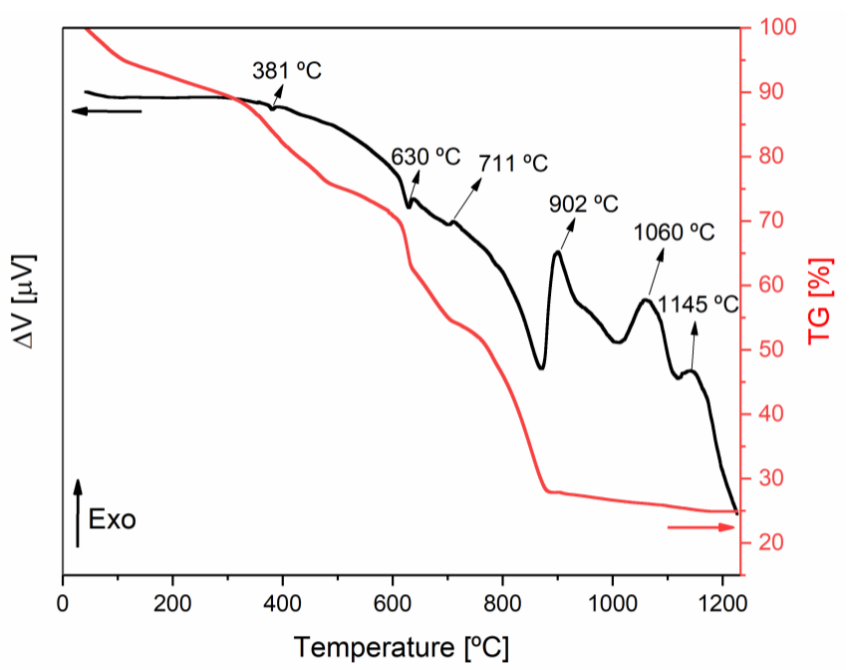
DTA/TGA thermograms obtained for the base powders. The black line is in respect of the DTA analysis, while the red is in respect of the TG. Arrows in the color of the lines point to the scale.

Figure 1 shows the DTA/TG results. It is possible to observe a loss of about 8% of the mass until a temperature of 200 °C, which is related to a loss of water, by the sample. Endothermic phenomena may be seen at temperatures of 381 and 630 °C, accompanied by a mass loss of 20%, approximately. These phenomena can be related to the removal of nitrates and the degradation of organic matter of the precursor of the reaction, respectively. Between 711 and 902 °C, another endothermic peak, accompanied by a 30% drop in mass, is observable. This phenomenon can also be related to the degradation of the organic matter in the precursor of the reaction since PCW has multiple organic compounds. At 711, 902, 1060, and 1145 °C, it is possible to observe exothermic phenomena without mass changes that might be related to the formation of new crystalline phases. Taking into account these results, heat treatments were performed at 350, 500, 800, 1000, and 1300 °C for 4 h, and this duration was supported by the literature [19]. Afterward, seeing that a pure crystalline potassium ferrite sample was not obtained, another heat treatment was performed by heating the powder at 600 °C for 24 h, forming a disk using a mould, and applying a uniaxial pressure (2.5 × 10^3^ N/mm^2^) and then heat-treated for 4 h at 1000 °C.

**Figure 2:**
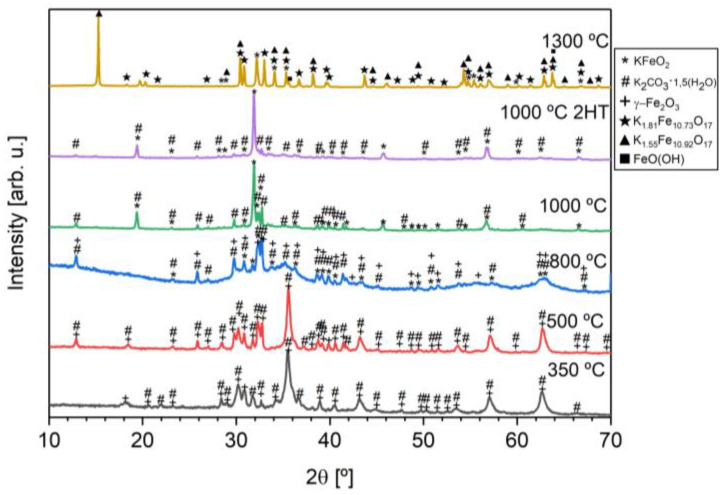
XRD diffractograms of the heat-treated samples.

Figure 2 shows the powder XRD diffractograms obtained for the samples with different heat treatments. Table 1 shows the phase’s composition and the amount present in each sample after heat treatment. Although their difference in percentages, heat-treated samples at 350, 500, and 800 °C, all have crystallographic phases compatible with maghemite (${\gamma -Fe}_{2}O_{3})$ and potassium carbonate ($K_{2}{CO}_{3}\cdot 1.5(H_{2}O)$). Raising the heat treatment temperature to 800 °C, it is possible to observe the existence of potassium carbonate and the formation of the potassium ferrite ($KFeO_{2})$ phase. This formation is consistent with the existence of the exothermic phenomenon at 711 °C obtained in the DTA. These two phases are present in the samples with heat treatments at 800, 1000, and 1000 °C with two heat treatments. The existence of potassium carbonate in these phases is consistent with what is reported in the literature [8] and is due to the caption of CO_2_ and H_2_O from the surrounding ambient air. This makes it difficult to obtain a pure phase without having a controlled environment. Increasing the temperature to 1300 °C, we see a decline in the desired phase (KFeO_2_), as well as the formation of transition phases, which was a reason to not characterize this sample further.

The sample heat treated at 1000 °C with two heat treatments has the highest percentage of $KFeO_{2}$ (83.3%). In opposition to some literature data on potassium ferrite [6], to obtain the potassium ferrite, there was a need for a heat treatment at the temperature of 800 °C, at least.

**Figure 3:**
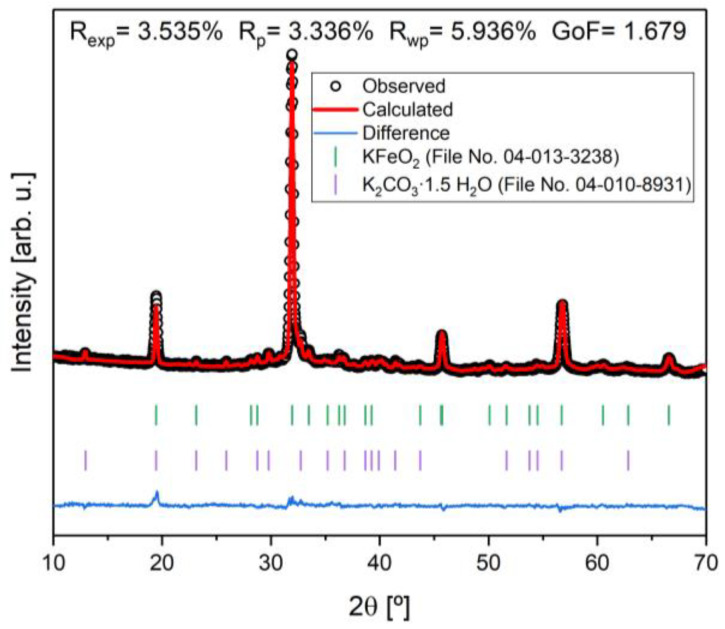
Rietveld refinement of the sample heat-treated at 1000 °C with 2 heat treatments and agreement indexes.

To further investigate the sample with the highest percentage of potassium ferrite, a Rietveld refinement was performed, as shown in Figure 3. Taking into account the fit, that R_wp_ ≥ R_exp_, that $\chi^{2}={\left(\frac{Rwp}{Rexp}\right)}^{2}$ is higher than 1, and that the goodness of fit (GoF), defined by GoF = $\sqrt{\chi^{2}}$, is close to one, it is revealed that the quality of the refinement is good [20,21].

**Figure 4:**
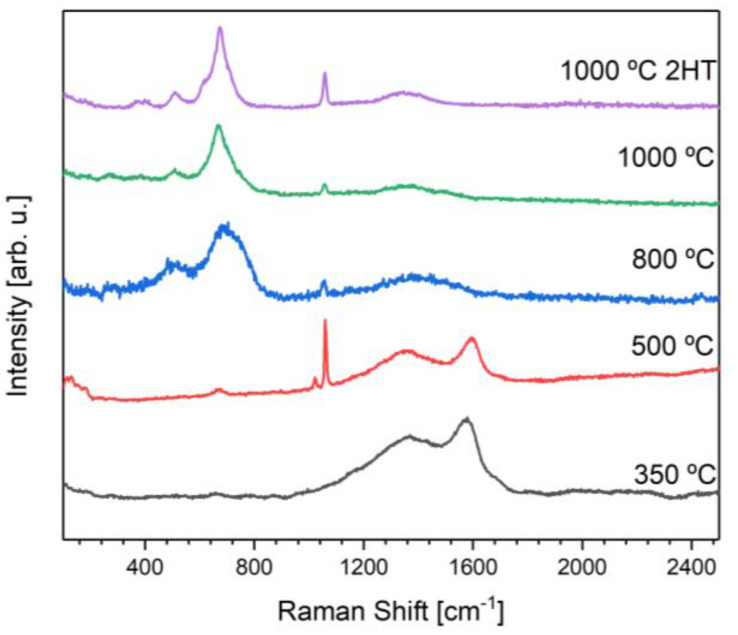
Raman spectra of the heat-treated samples.

XRD results are corroborated with Raman spectroscopy (Figure 4). When it comes to the samples containing $KFeO_{2}$, which are the ones with a heat treatment of at least 800 °C, all the samples share a vibrational band that might be attributed to the potassium ferrite (~508 ${cm}^{-1}$) [22]. Moreover, they also all share a vibrational band at ~1056 ${cm}^{-1}$ that might be associated with the presence of the carbonate ion [23]. For the samples with heat treatments at 350 and 500 °C, it is possible to observe two vibrational bands already reported in a maghemite Raman spectrum [24] at approximately 1360 and 1580 ${cm}^{-1}$. The vibrational bands and respective attribution may be seen in Table 2.

**Figure 5:**
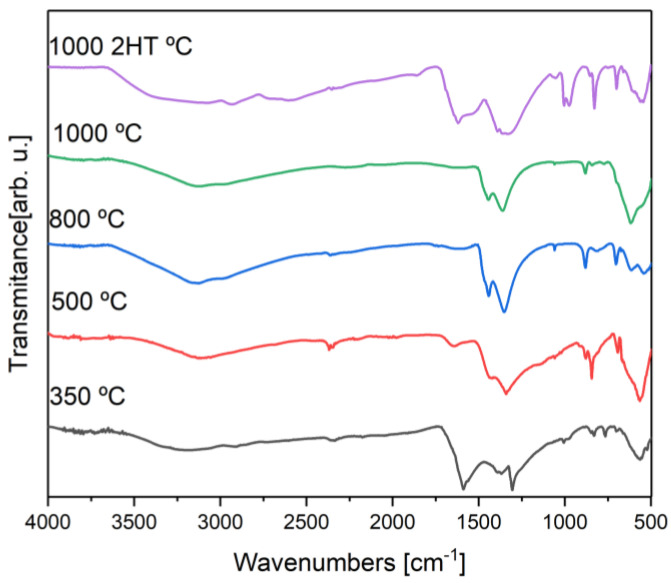
FTIR spectra of the heat-treated samples.

Figure 5 presents the FTIR spectra obtained for heat-treated samples. All samples have a vibration mode between 3205 and 3044 ${cm}^{-1}$, and it is attributed to the presence of the water hydroxide group, which may be free or absorbed by the sample [6]. Apart from the sample heat-treated at 1000 °C, the samples have a vibration mode between 2356 and 2344 ${cm}^{-1}$, attributed to atmospheric carbon dioxide due to the carbon dioxide present in the room where the assays were performed [25]. Corroborating the XRD results, between 1343 and 1367 ${cm}^{-1}$, a vibration mode is observed, which is credited to the C-O bonds in the carbonate ion [23]. Also corroborating the XRD results and Raman spectroscopy, in the samples with heat treatment of at least 800 °C, it is possible to observe a vibration mode between 1052 and 1060 ${cm}^{-1}$ attributed to the Fe-K bonds that are present in $KFeO_{2}$. The observed vibration modes and attribution are summarized in Table 3.

### 3.2. Morphological Analysis

**Figure 6:**
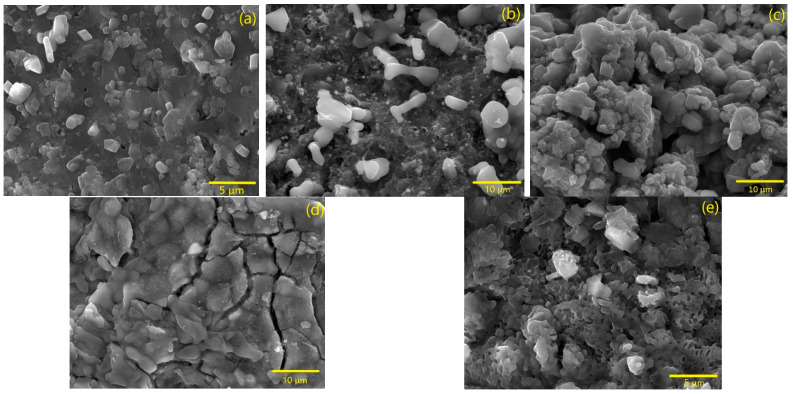
SEM micrographs for heat-treated samples at 350 °C (**a**), 500 °C (**b**), 800 °C (**c**), 1000 °C (**d**), and 1000 °C with 2HT (**e**).

The obtained micrographs for the samples with different heat treatments through an SEM analysis are presented in Figure 6. For the sample heat-treated at 350 °C, the existence of irregularly shaped grains is observed with an average size of 1.40 µm. The sample treated at 500 °C, which has the same phases as the sample heat-treated at 350 °C, the grain size augmented to 5.44 µm and a rod shape was observed, which is characteristic of maghemite [27]. In the sample treated at 800 °C, in which the KFeO_2_ phase is detected, by XRD results (Figure 2), a hexagonal grain habit characteristic of KFeO_2_ [28] is shown, corroborating the previous results. In addition, there is present an aggregation of grains and a decline in average grain size to 2.40 µm. For the other samples, the coalescence phenomenon is detectable, as the average grain size is much bigger than in the other samples.

### 3.3. Biological Analysis

**Figure 7:**
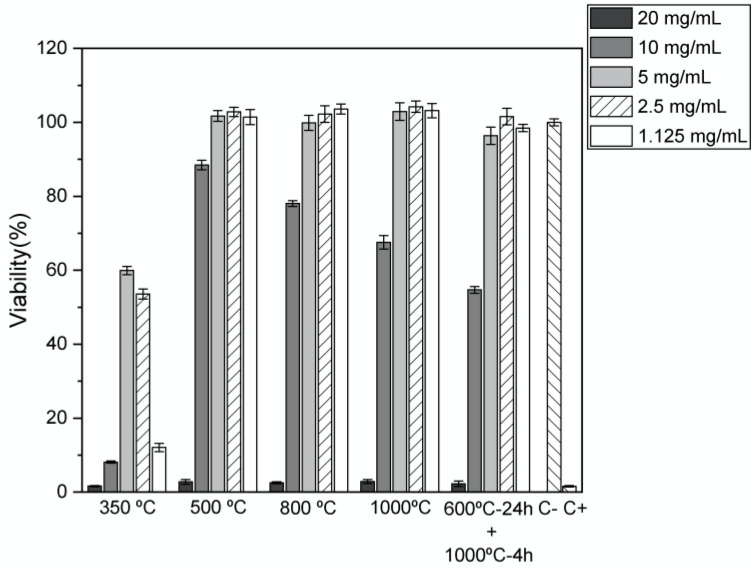
Relative cellular viability as a function of the heat treatment temperature for the samples heat treated at 350 °C, 500 °C, 800 °C, 1000 °C, and 600 °C + 1000 °C. The negative control is cells that are cultured in a non-cytotoxic environment, which is the reference for the relative populations calculation. When a relative population drops below 90%, that sample begins to display a varying degree of cytotoxicity.

Figure 7 reports the results of the cytotoxicity tests made in accordance to standard ISO-10993. For these tests to be relevant, they must reproduce, as much as possible, the operating conditions of a biomedical material [29,30,31]. The meaningful concentration range chosen for extract preparation was from 1.125 mg/mL to 20 mg/mL. The results show that for the highest concentration tested, all heat treatment methods result in cytotoxic materials. Heat treatment at 350 °C results in materials that are either moderately or severely cytotoxic. Excluding this sample, it is possible to observe that for concentrations until 5 mg/mL, the other samples are non-cytotoxic.

### 3.4. Magnetic Analysis

#### 3.4.1. VSM

**Figure 8:**
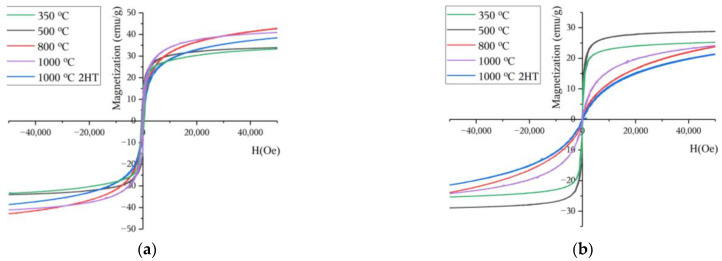
Magnetization curves obtained at (**a**) 5 K and (**b**) 300 K for the heat-treated samples.

For both temperatures used to measure the hysteresis cycle, 5 and 300 K (Figure 8), it is possible to see that all curves are close to saturation; hence, it is considered that within an uncertainty < 10%, the values of magnetization at 50 kOe are a good estimate for the saturation magnetization. In the samples heat-treated at 350, 500, and 800 °C, at 5 K, the estimated saturation magnetizations were 33.3, 33.9, and 42.8 emu/g, respectively. At 300 K, they were 25.2, 28.2, and 23.74 emu/g, respectively. For these three samples, XRD characterization suggests the presence of a $\gamma -{Fe}_{2}O_{3}$ phase. According to the literature, this phase presents a saturation magnetization of 74 emu/g [32] at 300 K, and this is a way to explain the high magnetization of these samples compared to others, considering the diamagnetic behaviour of potassium carbonate (χ = −59.0 × 10^−6^ cm^3^/mol) [33]. The samples heat treated at 1000 °C also presents a high magnetization, suggesting that potassium ferrite will also have a high magnetization.

Using the SQUID equipment, it was possible to more precisely assess the hysteresis cycle area, focusing on the region of H = 0 Oe.

#### 3.4.2. SQUID

**Figure 9:**
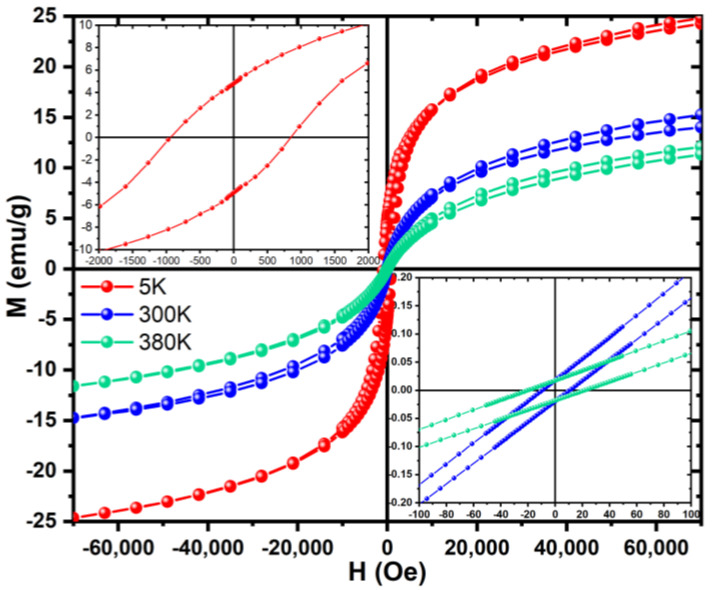
Magnetization Curves for the two heat treatments samples at 5, 300, and 380 K.

To further analyze the magnetization curve of the sample that had the biggest percentage of potassium ferrite, meaning the sample heat-treated at 1000 °C with 2 heat treatments, a SQUID was used. Hysteresis cycles were obtained at the temperatures of 5, 300, and 380 K, as observed in Figure 9. From this figure, it is clear that the area of the hysteresis cycles is not null. In this manner, this can be one of the heat dissipation mechanisms of these particles. This shows, as expected, that the particles are not superparamagnetic once the grain size is micrometric, not agreeing with the suggestions of some of the literature for this crystal phase [5].

#### 3.4.3. MHT

The obtained results from MHT measurements are listed in Table 4.

As it is possible to observe, the samples with heat treatments at 350 and 500 °C do not show evidence of having potassium ferrite in their composition with X-Ray diffraction and Raman spectroscopy and have a bigger SAR when compared with the other samples. This may have happened since these two samples have maghemite in their composition. If these particles had a size between 5 and 100 nm, they would have an adequate profile for application in magnetic hyperthermia [5]. Using this method of synthesis, microparticles were obtained, and, due to their size, they cannot be applied in magnetic hyperthermia.

Heat may be dissipated by hysteresis, Brownian and Néel relaxation, and by eddy currents caused by friction and viscous suspensions [19]. Since the particles have micrometric size, as they are too big to exist with influenceable Brownian and Néel relaxation, it is predicted that the heat dissipation is due to magnetic hysteresis. For the samples heat treated at 350 and 500 °C, it is possible to observe that they have a bigger SAR than the other samples. These two samples will achieve a much higher magnetization than other samples for small applied fields. In this way, it is possible to obtain a bigger induced electromotive force with a low-intensity external magnetic field applied [19], translating into higher SAR values.

The use of potassium ferrite for magnetic hyperthermia should not yet be discarded, as SAR is dependent on particle size, meaning that smaller-sized particles could have a bigger SAR, appropriate for magnetic hyperthermia [34].

## 4. Conclusions

Using powdered coconut water as a precursor for a sol-gel reaction, the synthesis of potassium ferrite (KFeO_2_) was achieved for temperatures above 800 °C, although the highest percentages were observed in the samples with heat treatments at 1000 °C. The sample with two heat treatment plateaus (600 °C for 24 h and 1000 °C for 4 h) presented the highest percentage of KFeO_2_, portraying 83.3% of the sample. In this work, a controlled atmosphere was not used, meaning that a pure phase sample is difficult to obtain since the exposure to ambient air will produce K_2_CO_3_·1.5H_2_O. Through SEM, it was possible to observe that microparticles were obtained. This size is incompatible with employment in cancer treatment through magnetic hyperthermia. The samples containing potassium ferrite were non-cytotoxic for extract concentrations up to 5 mg/mL and can, therefore, be safely used in biomedical applications—insofar as a cytotoxicity test can assess, in a limited way, material biocompatibility. The fact that the obtained microparticles were non-cytotoxic for high concentrations, suggests that if nanoparticles were synthesized they would be even less cytotoxic.

The heat dissipation mechanism for these samples was credited to hysteresis loss effects (magnetic domain creation and destruction). The obtained SAR for the potassium ferrite samples was low, but this material should not yet be discarded for magnetic hyperthermia, as its SAR can be quite different if one explores its performance in a superparamagnetic regime. In order to explore the full potential of this material, nanoparticles should be obtained and studied. Some possible routes for this objective include the modification or change of the used synthesis method or the use of ball milling to reduce the size of the particles obtained in this work.

## Figures and Tables

**Table 1:** Crystalline phases and quantification for the heat-treated samples.

Heat Treatment Temperature (°C)	Crystalline Phase and Composition (%)
350	${\gamma -Fe}_{2}O_{3}$ (98.5%) $K_{2}{CO}_{3}\cdot 1.5(H_{2}O)$ (1.5%)
500	${\gamma -Fe}_{2}O_{3}$ (58.4%) $K_{2}{CO}_{3}\cdot 1.5(H_{2}O)$ (41.6%)
800	${\gamma -Fe}_{2}O_{3}$ (36.9%) $KFeO_{2}$ (12.9%) $K_{2}{CO}_{3}\cdot 1.5(H_{2}O)$ (50.2%)
1000	$KFeO_{2}$ (55.1%) $K_{2}{CO}_{3}\cdot 1.5(H_{2}O)$ (44.9%)
1000 2HT	$KFeO_{2}$ (83.3%) $K_{2}{CO}_{3}\cdot 1.5(H_{2}O)$ (16.7%)
1300	$KFeO_{2}$ (30%) $K_{1.81}{Fe}_{10.73}O_{17}$ (24%) $K_{1.55}{Fe}_{10.92}O_{17}$ (35%) $FeO\left(OH\right)$ (11%)

**Table 2:** Raman shift attributions to CO_3_^2−^ [23], ${\gamma -Fe}_{2}O_{3}$ [24], and $KFeO_{2}$ [22].

	350 °C	500 °C	800 °C	1000 °C	1000 °C 2HT	Attribution
$\mathrm{Vibrational}\;\mathrm{Bands}\;({cm}^{-1}$)			268	273		$KFeO_{2}$
					395	$KFeO_{2}$
			508	509	508	$KFeO_{2}$
	656	670	682	667	673	$CO_{3}{}^{2-}$, ${\gamma -Fe}_{2}O_{3}$ or $KFeO_{2}$
		1021				Not identified
		1059	1056	1056	1058	$CO_{3}{}^{2-}$
	1370	1351	1386	1373	1360	$CO_{3}{}^{2-}$ or ${\gamma -Fe}_{2}O_{3}$
	1578	1596				${\gamma -Fe}_{2}O_{3}$

**Table 3:** Table with the vibration modes obtained for the heat-treated samples and respective attribution.

HT Temp. (°C)	Vibration Modes (cm^−1^)														
1000 (2HT)	3056	2930	2352	1620		1338	1052		980		828		700		548
1000	3137				1442	1362	1059			878	842	769		618	
800	3143		2356		1442	1351	1060			881			702	615	537
500	3119		2355	1650		1343				878	844		692		564
350	3205		2344	1591		1367		1004			830	767	700		562
Atributtion	O-H [6]	$CO_{2}$ atm. [25]	$CO_{2}$ atm. [25]	$H_{2}O$ [26]		C-O [23]	Fe-K [6]		Fe-K [6]	C-O [23]	Fe-O [6]		$\left(CO_{3}{}^{2-}\right)$ [23]	M-O [10]	M-O [10]

**Table 4:** Table with the temperature variation and SAR obtained for the heat-treated samples.

Heat Treatment (°C)	ΔT (°C); [0; 650] s	SAR (W/g)
350	16.8 ± 1.01	24.2 ± 3.58
500	18.0 ± 0.15	21.8 ± 4.29
800	2.67 ± 0.68	2.65 ± 1.21
1000	3.93 ± 0.29	5.76 ± 0.96
1000 (2HT)	1.97 ± 0.46	1.55 ± 0.06

## Data Availability

Not applicable.
